# Multifunctional Gelatin‐Based Smart Films Integrating Thermochromic Encryption, Temperature‐Regulated Photothermal Management, Reprocessability, and Biodegradability for Sustainable Applications

**DOI:** 10.1002/advs.202516955

**Published:** 2026-01-04

**Authors:** Yuehong Zhang, Chen Yang, Langlang Dai, Leipeng Liu, Vijay Kumar Thakur

**Affiliations:** ^1^ College of Bioresources Chemical and Materials Engineering Shaanxi University of Science and Technology Xi'an China; ^2^ School of Chemical Engineering and Technology Tianjin University Tianjin China; ^3^ Biorefining and Advanced Materials Research Centre Scotland's Rural College Edinburgh Scotland UK

**Keywords:** information encryption, multifunctional gelatin films, photothermal regulation, sustainable smart materials, temperature regulation, thermochromic materials

## Abstract

Multifunctional gelatin‐based smart films are engineered by incorporating hyperbranched polyglycerol (HBPG) as a plasticizer, dialdehyde β‐cyclodextrin (Da‐β‐CD) as a crosslinker, and thermochromic microcapsules (TCMs). Structural analyses, including FTIR, XPS, and NMR, confirm the formation of covalent Schiff base linkages between Da‐β‐CD and gelatin, alongside hydrogen bonding reorganization facilitated by HBPG. The optimized film (GHBT_2_‐CD) exhibits enhanced tensile strength (28.7 MPa), hydrophobicity (water contact angle of 116°), UV‐blocking capability (>97%), and complete (100%) bacterial inhibition. Crucially, these films demonstrate programmable thermochromism for multilevel information encryption, enabling features such as laser‐writing, temperature‐gated message display (e.g., “SUST” decryption), and numeral switching (9→7→8) using TCMs with distinct transition temperatures (18°C, 28°C, and 38°C). Furthermore, they achieve dual‐modal encryption by combining the intrinsic fluorescence of gelatin (emission at 340 nm) with thermochromism, which enables four‐state displays (e.g., showing “Accept”). Additionally, the films provide self‐adaptive temperature regulation: their black state below 28°C significantly boosts solar heating (achieving a ΔT of +27°C in a 4°C ambient environment), while their pale‐yellow state above 28°C mitigates overheating (keeping the surface below 56°C in a 30°C ambient), an effect augmented by the phase‐change latent heat buffering of the TCMs. Finally, the films embody closed‐loop sustainability. The presence of dynamic Schiff base and hydrogen bond networks enables over 91% self‐healing efficiency using stimuli like water, heat, or vapor, facilitates physical reprocessing, and allows for tunable degradation rates dependent on pH or soil conditions (complete degradation within 24 h at pH = 2, and within 12 days in sludge). This work pioneers an all‐in‐one smart materials platform that bridges optical security, thermal management, and the principles of a circular economy.

## Graphical abstract

Multifunctional gelatin films synergizing thermochromic encryption, self‐regulating photothermal management, and closed‐loop recyclability offer sustainable solutions for intelligent packaging.

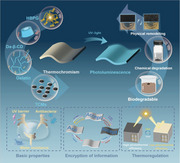

## 1 Introduction

The widespread use of traditional plastic films, notably polyethylene (PE) and polypropylene (PP), is driven by their cost‐effectiveness and robust performance [1, 2]. However, their petroleum‐derived, non‐biodegradable nature perpetuates severe ecological crises through persistent microplastic pollution, posing significant threats to ecosystems and human health [3, 4, 5]. This urgent challenge necessitates the development of sustainable, high‐performance bio‐based alternatives. Gelatin, a natural biopolymer extracted from collagen‐rich tissues such as skin, bones, and tendons, emerges as a prime candidate due to its inherent biodegradability, ease of forming high‐solid‐content aqueous solutions, excellent film‐forming ability, biocompatibility, and low toxicity [6, 7, 8]. These attributes underpin its established applications in biomedicine [9, 10, 11] and food packaging [12, 13], positioning it as an ideal candidate for sustainable films. Despite these advantages, native gelatin films suffer from critical limitations, including poor mechanical strength and susceptibility to moisture, which severely restrict their commercial viability.

Strategies such as chemical cross‐linking [14, 15], polymer blending [16, 17], nanocomposite reinforcement [18, 19], and plasticization [20, 21] (e.g., with glycerol) offer partial improvements but often compromise self‐healing capability or fail to impart advanced functionalities. For instance, Wei et al. [22] employed an in situ anthocyanin crosslinking approach to simultaneously enhance the mechanical strength, moisture barrier, and optical properties of gelatin films. Similarly, Shiv et al. [23] demonstrated that incorporating melanin nanoparticles significantly improved both mechanical integrity and thermal stability. However, irreversible covalent cross‐linking, while capable of substantially reinforcing mechanical strength, inevitably suppresses the dynamic reconfigurability of the network, thereby leading to a loss of self‐healing capability [24, 25]. Meanwhile, conventional plasticizers like glycerol enhance flexibility but can significantly degrade mechanical strength at higher loadings [26]. Overall, existing modifications often sacrifice critical properties and lack the advanced functionalities required for emerging applications, such as intelligent packaging that demands optical security or adaptive thermal regulation.

Thermochromic microcapsules (TCMs), which are capable of reversible color changes in response to temperature, hold immense promise for such smart applications. Organic TCMs, in particular, offer advantages including tunable transition temperatures, high sensitivity, and cost‐effectiveness compared to inorganic or liquid crystal counterparts [27, 28, 29]. Their integration into films could enable valuable temperature‐responsive optical signaling for encryption and solar‐energy modulation for thermoregulation. However, incorporating TCMs typically degrades mechanical properties due to poor matrix compatibility [30], creating a fundamental tension between achieving functionality and maintaining performance. Currently, no strategy successfully integrates thermochromism with robust mechanics, self‐healing capability, recyclability, and multi‐modal encryption within a single, sustainable gelatin platform.

**FIGURE 1:**
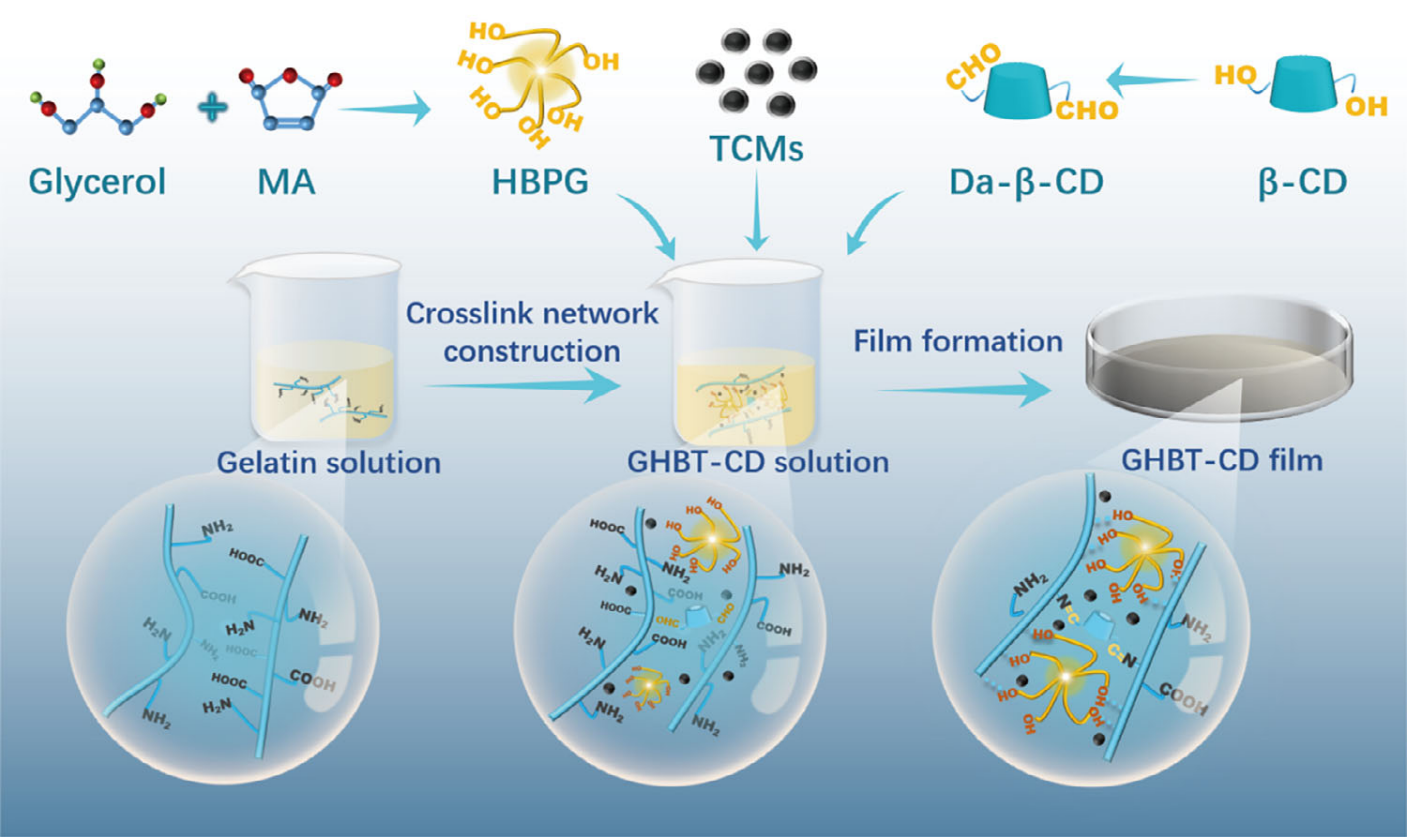
Schematic illustration of the preparation process for multifunctional gelatin‐based films modified with Da‐β‐CD, HBPG, and TCMs.

Herein, we present a transformative material design that overcomes these limitations: multifunctional gelatin‐based films synergizing thermochromic encryption, self‐adaptive thermal regulation, and closed‐loop sustainability (Figure 1). Our strategy employs TCMs for programmable color switching; sustainably synthesized bio‐based hyperbranched polyglycerol (HBPG) from glycerol and maleic anhydride as a superior plasticizer (replacing small‐molecule glycerol) whose abundant hydroxyl groups form dense, toughening hydrogen bonding networks with gelatin; and dialdehyde β‐cyclodextrin (Da‐β‐CD) as a covalent crosslinker that forms mechanically reinforcing and fluorescent Schiff base bonds with gelatin's amino groups while also imparting antimicrobial activity [31, 32].

Crucially, the dynamic nature of these Schiff bases and hydrogen bonds unlocks unprecedented sustainability features, enabling stimulus‐responsive self‐healing, physical reprocessability, and pH‐tunable degradation. In this work, we demonstrate how this engineered system achieves multilevel information encryption via dual‐modal (fluorescence/thermochromism) displays and temperature‐gated decryption; intelligent thermoregulation through autonomous solar‐heating in the black state below 28°C and heat‐rejection in the pale‐yellow state above 28°C, buffered by the TCMs' phase‐change latent heat; and a circular lifecycle supporting closed‐loop recycling. This work introduces a new eco‐friendly material intended for next‐generation sustainable applications in anti‐counterfeiting, smart thermal regulation, and environmental protection.

## 2 Results and Discussion

### 2.1 Structural Analysis of Da‐β‐CD, HBPG and Modified Gelatin‐Based Films

**FIGURE 2:**
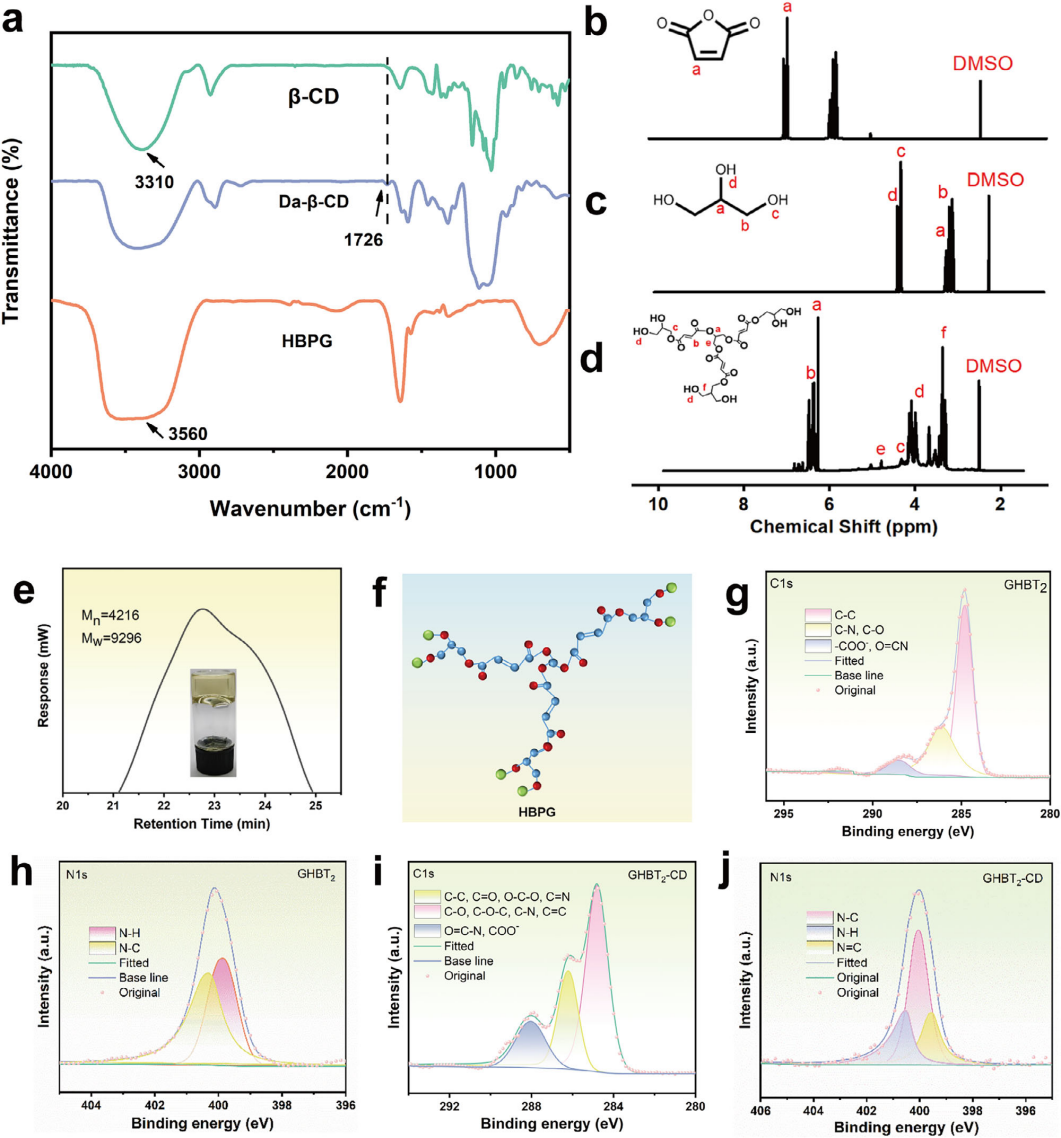
Structural characterization: a) FTIR spectra of TCMs, β‐CD, and Da‐β‐CD. b–d) ^1^H NMR spectra of maleic anhydride, glycerol, and synthesized HBPG. e) Molecular weight distribution of HBPG determined by GPC. f) Ball‐and‐stick model depicting the structure of HBP. g, h) High‐resolution XPS C 1s and N 1s spectra of the GHBT_2_ film (gelatin/HBPG/TCMs). i, j) High‐resolution XPS C 1s and N 1s spectra of the GHBT_2_‐CD film (gelatin/HBPG/TCMs/Da‐β‐CD).

Fourier‐transform infrared (FTIR) spectroscopy confirmed the structural changes upon oxidation of β‐cyclodextrin (β‐CD) to dialdehyde β‐cyclodextrin (Da‐β‐CD). As shown in Figure 2a and Figure S2a, the characteristic peak corresponding to hydroxyl group (─OH) stretching vibrations in β‐CD was observed at 3310 cm^−1^. In contrast, the spectrum of Da‐β‐CD exhibited a new peak at 1726 cm^−1^, attributed to the C═O stretching vibration of the aldehyde group. The appearance of this aldehyde group confirms the successful oxidation of hydroxyl groups in β‐CD to form aldehyde groups in Da‐β‐CD, consistent with previous reports on β‐cyclodextrin oxidation [33]. To further confirm the structural modification of β‐cyclodextrin, the aldehyde content in Da‐β‐CD was quantified at 6.61 mmol/g using a hydroxylamine hydrochloride titration assay (Figure S2b). This confirms the successful introduction of aldehyde groups. Furthermore, Further evidence was provided by ^1^H‐NMR spectra (Figure S2c). The spectra of Da‐β‐CD exhibit a new proton signal at 9.5 ppm, corresponding to the aldehyde hydrogen, which is absent in native β‐CD. The relatively low intensity of this signal is likely due to hemiacetal formation. Consistent with this, additional peaks observed at 7.0 and 6.5 ppm can be attributed to protons in the hemiacetal form.

The structural characteristics of the synthesized hyperbranched polyglycerol (HBPG) plasticizer were systematically investigated using ^1^H NMR spectroscopy (Figure 2b–d). Peaks at 4.42 and 4.5 ppm, attributed to the hydroxyl protons of glycerol, disappeared after reaction with maleic anhydride. Simultaneously, a new peak corresponding to terminal hydroxyl groups appeared at 3.9 ppm, and a new methylene peak adjacent to the ester group appeared at 4.32 ppm, confirming the synthesis of HBPG. This successful preparation was further corroborated by FTIR analysis (Figure 2a), which showed characteristic absorption peaks for terminal hydroxyl groups at 3560 cm^−1^. Gel permeation chromatography (GPC) analysis (Figure 2e) revealed that HBPG had a number‐average molecular weight (M_n_) of 4216 Da, a weight‐average molecular weight (M_w_) of 9296 Da, and a polydispersity index (PDI) of 2.2, confirming its synthesis from glycerol and maleic anhydride.

XPS analysis was performed on GHBT_2_ and GHBT_2_‐CD films to investigate the covalent crosslinking interaction between Da‐β‐CD and gelatin. The C 1s spectra of the GHBT_2_ film (Figure 2g) displayed characteristic peaks at 284.8 eV (C─C), 286.1 eV (C─O & C─N, and 288.9 eV (O═C─N & ─COO─), consistent with the known structure of gelatin [34, 35]. Following the introduction of Da‐β‐CD, the proportion of the C‐O/C‐N component in the C 1s spectra (Figure 2i) increased significantly from 28.53% in GHBT_2_ films to 35.28% in the GHBT_2_‐CD film. This increase indicates covalent cross‐linking between the aldehyde group of Da‐β‐CD and the amino group of gelatin. A corresponding increase in the proportion of C─N bonds was also observed in the N 1s spectra (Figure 2h,j; Table S1), confirming the formation of new C─N covalent bonds attributable to the Schiff base reaction between Da‐β‐CD and gelatin.

Further investigation of the modified gelatin‐based films using FTIR spectroscopy (Figure S1) provided additional evidence for structural interactions. The GT_2_ film (gelatin and glycerol) exhibited characteristic peaks at approximately 3300 cm^−1^ (N─H stretching), 1639 cm^−1^ (amide I, C═O stretching), 1535 cm^−1^ (amide II, C─N stretching and N─H bending), and 1236 cm^−1^ (amide III, C─N and N─H vibration). Upon addition of HBPG, the C─N stretching vibration peak shifted from 1535 to 1546 cm^−1^, and the C─N and N─H stretching vibration peak shifted to 1222 cm^−1^. These shifts are attributed to supramolecular interactions, specifically hydrogen bonding, between the terminal hydroxyl groups of HBPG and the ─CONH and ─OH groups of gelatins, which partially restrict the vibrational freedom of the peptide bonds [36]. Upon incorporation of Da‐β‐CD, the characteristic symmetric stretching peak of the aldehyde group at 1725 cm^−1^ disappeared, while the C─N stretching vibration peak shifted further from 1546 to 1556 cm^−1^, indicative of Schiff base bond formation. Concurrently, the amide I peak (originally at 1639 cm^−1^) shifted to 1647 cm^−1^, and the intensity of the N─H peak at 3300 cm^−1^ decreased, attributed to a reduction in N‐H groups [32]. These FTIR spectral changes confirm the formation of hydrogen bonds between HBPG and gelatin, as well as covalent crosslinking between Da‐β‐CD and gelatin via Schiff base formation.

### 2.2 Basic Properties of Modified Gelatin‐Based Films

**FIGURE 3:**
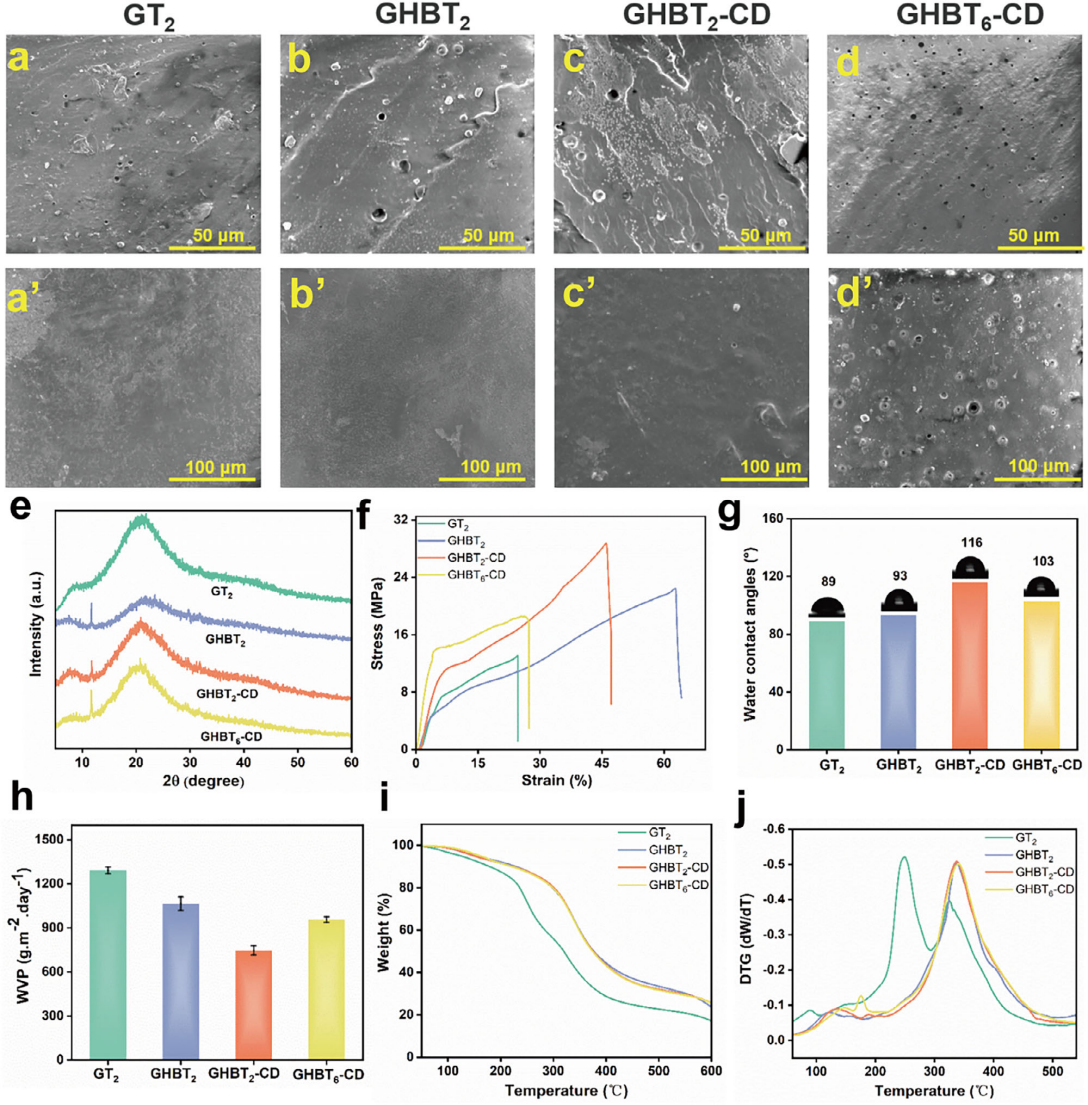
Morphological, structural, and fundamental properties: a–d) Cross‐sectional and a'–d') planar SEM images of GT_2_ (gelatin/glycerol/TCMs), GHBT_2_ (gelatin/HBPG/TCMs), GHBT_2_‐CD (gelatin/HBPG/TCM/Da‐β‐CD), and GHBT_6_‐CD (gelatin/HBPG/higher TCM/Da‐β‐CD) films. e) X‐ray diffraction (XRD) patterns. f) Tensile strength and elongation at break. g) Water contact angles. h) Water vapor transmission rates. i) Thermogravimetric analysis (TGA) curves. j) Derivative thermogravimetry (DTG) curves.

SEM revealed distinct morphological differences between the films. Cross‐sectional images (Figure 3a–d) show the pore defect structures in the GT_2_, GHBT_2_, GHBT_2_‐CD, and GHBT_6_‐CD films, attributed to the presence of TCMs. Compared to the relatively smooth cross‐section of the GT_2_ film, the GHBT_2_ film exhibited a dense and uniform layered structure, resulting from an extensive hydrogen bonding network formed between HBPG and gelatin, indicating enhanced fracture resistance. After cross‐linking with Da‐β‐CD, the GHBT_2_‐CD film displayed a lamellar fracture morphology attributable to strengthened intermolecular forces from the covalent crosslinking network between Da‐β‐CD and gelatin, which increases rigidity but reduces flexibility. As shown in Figure S3, the TCMs exhibit a narrow size distribution of 2–5 µm. Nevertheless, when the TCMs content reaches 6% (GHBT_6_‐CD film), abundant micropores are generated, severely disrupting the film's internal structure and consequently compromising both its rigidity and elongation at break. Surface morphology analysis (Figure 3a'–d') showed that GT_2_ and GHBT_2_ films possessed smooth surfaces, reflecting homogeneous mixing and strong interfacial compatibility between HBPG and gelatin. The surface remained smooth after Da‐β‐CD incorporation, confirming its uniform dispersion. The introduction of TCMs increased surface roughness, with significant enhancement at 6% loading.

XRD spectroscopy (Figure 3e) verified the crystalline structure of the gelatin‐based films. The GT_2_ film exhibited characteristic diffraction peaks at 2θ≈ 8° (crystalline domains of collagen‐like triple helices) and 20° (amorphous region) [37]. The introduction of HBPG significantly reduced the peak intensity at 2θ ≈ 8°, indicating that the hydrogen bond network formed between HBPG and gelatin disrupts molecular chain ordering and diminishes crystallinity [38, 39]. For the GHBT_2_‐CD film (5% Da‐β‐CD), peak intensity at 2θ ≈ 8° decreased while intensity at 2θ ≈ 21° increased, suggesting that chemical cross‐linking reduces crystalline domains but enhances the flexible amorphous region [40]. Higher TCMs content further reduced crystallinity in the GHBT_2_‐CD film.

Mechanical properties critically determine the applicability of gelatin‐based films (Figure 3f). Replacing conventional glycerol with hyperbranched HBPG significantly enhanced properties: the GHBT_2_ films (gelatin/HBPG/TCM) achieved a 75% increase in tensile strength (22.4 MPa) and a 160% increase in elongation at break (63%) compared to the GT_2_ film (gelatin/glycerol/TCMs; tensile strength: 12.8 MPa; elongation at break: 24.1%). This substantial improvement stems from the dense hydrogen bond network formed between the abundant terminal hydroxyl groups of HBPG and functional groups (─NH_2_, ─OH, ─COOH, amide) of gelatin, which restricts chain slippage and improves tensile strength. Simultaneously, the reversible dissociation of hydrogen bonds acts as a sacrificial bond for energy dissipation, while the branched structure of HBPG expands free volume, reducing intermolecular forces and improving toughness. Strong interfacial bonding between gelatin and HBPG further enhances toughness by enabling efficient stress transfer [41]. Da‐β‐CD cross‐linking further increased tensile strength (28.7 MPa) but reduced elongation at break (46.1%) in the GHBT_2_‐CD film due to restricted chain mobility within the crosslinking network. Increasing TCMs content to 6% degraded both tensile strength and elongation at break owing to poor dispersion and weak TCM‐gelatin interactions.

Surface hydrophobicity was evaluated using static water contact angle (WCA) measurements (Figure 3g). WCAs for GT_2_ and GHBT_2_ films were 89° and 93°, respectively. The GHBT_2_‐CD film exhibits a significantly higher WCA of 116°, as Schiff base reactions consume hydrophilic ─NH_2_ groups of gelatins and increase the cross‐linking density. The GHBT_6_‐CD showed a lower WCA than GHBT_2_‐CD due to excess hydrophilic TCMs.

Water vapor permeability (WVP) is a key indicator for assessing barrier properties in food packaging (Figure 3h) [42, 43]. The GT_2_ film had a WVP of 1.292×10^3^ g m^−2^·day^−1^. Introducing HBPG decreased the WVP for the GHBT_2_ films to 1.065×10^3^ g m^−2^ day^−1^ by hindering vapor diffusion via the hydrogen bonding network. Da‐β‐CD incorporation further reduced WVP due to extended diffusion paths through the rigid glucose structural units of Da‐β‐CD and the formation of a chemical crosslinking network [44] The GHBT_6_‐CD film showed higher WVP than GHBT_2_‐CD because of excess hydrophilic TCMs.

Thermogravimetric analysis (TGA) assessed the thermal stability of the films (Figure 3i,j). All modified gelatin‐based films exhibited similar three‐stage mass loss profiles. However, significant differences were observed in both the maximum degradation temperatures (*T*_max_) and residual carbon contents. Specifically, GHBT_2_, GHBT_2_‐CD, and GHBT_6_‐CD films displayed higher *T*_max_ values (332°C, 335°C, 341°C) and greater residual carbon contents (23.6%, 25.6%, 25.5%)compared to the GT_2_ film (249°C, 17.1%), demonstrating that the incorporation of HBPG and Da‐β‐CD effectively enhances thermal stability.

### 2.3 Antimicrobial and UV‐Blocking Properties of Modified Gelatin‐Based Films

To elucidate the antimicrobial mechanism of the modified gelatin films, a multi‐faceted evaluation system was established, encompassing bactericidal potency, cellular viability, structural integrity, surface charge modulation, and biofilm inhibition.

The minimum bactericidal concentration (MBC) was first determined. Plate counting revealed that the films achieved a bactericidal rate of ≥99.9% against *E. coli* and *S. aureus* at concentrations of 10.5 and 14.0 mg/mL, respectively (Figure S4a,b), confirming their potent bactericidal efficacy. Direct bactericidal assays further underscored the critical role of Da‐β‐CD. While the control films (GT_2_ and GHBT_2_) showed negligible to moderate activity, both GHBT_2_‐CD and GHBT_6_‐CD achieved complete bacterial eradication (100%). This is attributed to the reactive aldehyde groups introduced by Da‐β‐CD, which interact with bacterial surface components.

**FIGURE 4:**
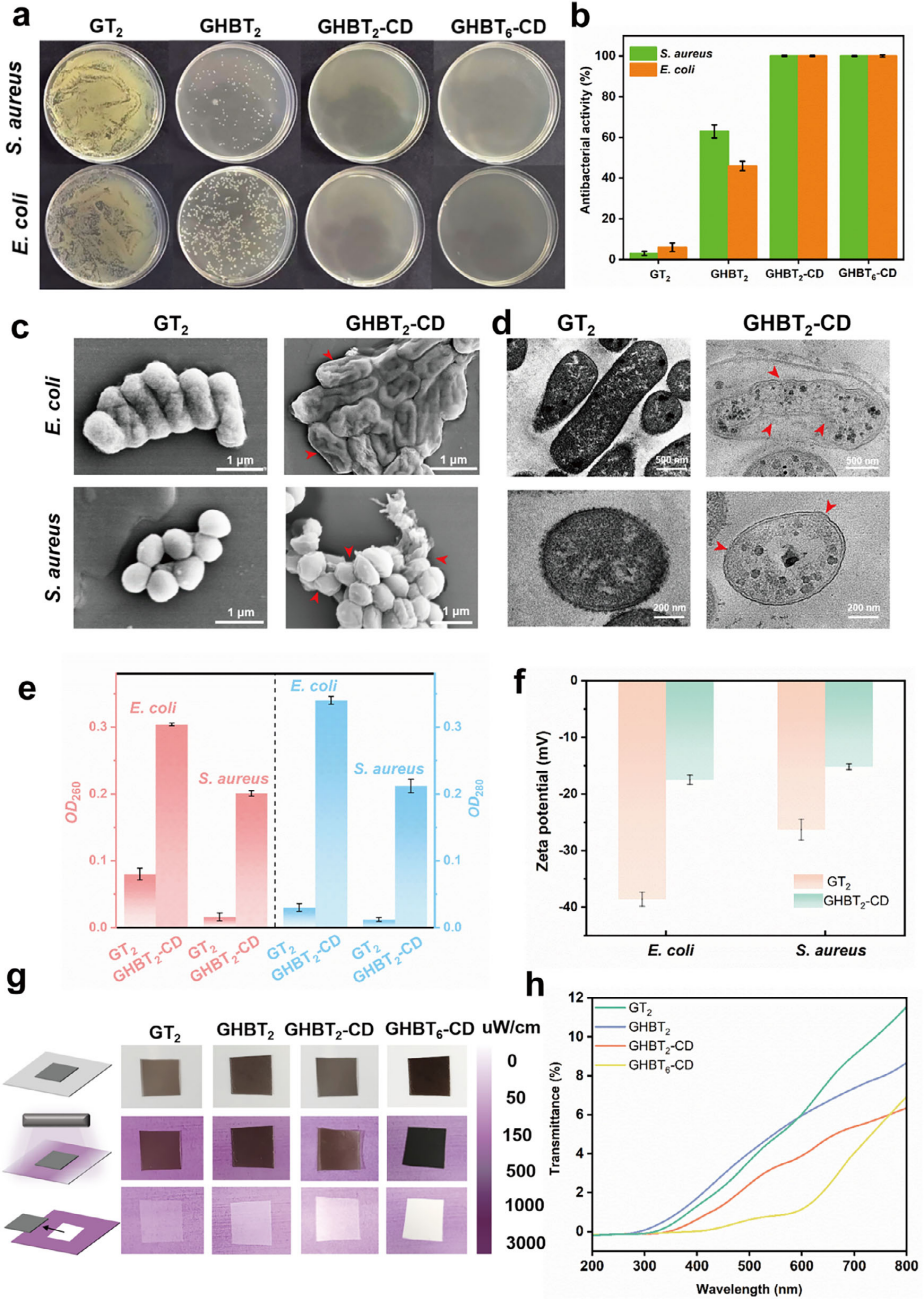
Functional properties: a) Photographs demonstrating the antimicrobial activity of films against *E. coli* and *S. aureus* after 24 h incubation. b) Corresponding quantitative bacterial inhibition rates. c) SEM and d) TEM images of bacterial cells after exposure to GT_2_ and GHBT_2_‐CD films. e) Quantitative analysis of bacterial membrane permeability after film treatment. f) Zeta potential of bacteria after film exposure.g) Optical photographs showing the color change of UV‐sensitive cards covered with different films after UV irradiation, indicating UV‐blocking efficacy. h) UV–vis transmittance spectra of the films across the 200–800 nm range.

Morphological changes were visualized by electron microscopy. SEM images (Figure 4c) showed that untreated bacteria maintained smooth, intact surfaces with characteristic shapes. In contrast, treatment with GHBT_2_‐CD caused severe structural damage, including cell shrinkage, collapse, and envelope rupture [45, 46]. Complementing these observations, TEM analysis (Figure 4d) revealed obvious disruption of the cell membrane and wall, leading to content leakage and a hollowed morphology, corroborating the membrane damage seen by SEM [47]. To quantitatively assess membrane integrity, bacterial membrane permeability was evaluated (Figure 4e). Treatment with GHBT_2_‐CD induced a significant increase in the release of nucleic acids and proteins (absorbance at 260 and 280 nm), confirming membrane disruption and intracellular content leakage [48]. Furthermore, zeta potential measurements (Figure 4f) indicated that bacterial surfaces became less electronegative after exposure to GHBT_2_‐CD compared to GT_2_, suggesting interactions between film aldehyde groups and surface components that may exacerbate membrane dysfunction [49]. Finally, the films' ability to suppress biofilm formation was assessed. All modified films (GHBT_2_, GHBT_2_‐CD, GHBT_6_‐CD) showed obvious biofilm inhibition compared to GT_2_ (Figure S5a). Quantitative analysis (Figure S5b) further revealed that GHBT_2_‐CD reduced the biofilm mass of *E. coli* and *S. aureus* by 65.2% and 54.1%, respectively, after 24 h (Figure S5b). In comparison, GHBT_2_ showed relatively weaker biofilm inhibition, which is consistent with the bactericidal assay results.

Collectively, these results demonstrate that GHBT_2_‐CD exerts a robust and multifaceted antimicrobial effect. The mechanism primarily involves aldehyde‐mediated interactions with the bacterial surface, leading to membrane disruption, loss of structural integrity, and suppression of biofilm formation, effectively targeting both *E. coli* and *S. aureus*.

All modified gelatin‐based films demonstrated substantial UV‐blocking capability across 200–400 nm (Figure 4g). Both GT_2_ and GHBT_2_ films blocked >95% of UVC (200–280 nm) and UVB (280–315 nm) radiation. The GHBT_2_‐CD film showed enhanced performance with 97% UV blocking across the full UV spectrum (200–400 nm, including UVA). This improvement is due to UV absorption by Schiff base bonds formed during Da‐β‐CD‐gelatin cross‐linking [50]. The GHBT_6_‐CD film achieved near‐complete UV blocking (100%), primarily owing to the strong absorption of the black dye within the TCMs, which effectively reduces transmittance [51]. These spectral findings were corroborated visually using UV‐sensitive cards (Figure 4g,h). Cards covered by GT_2_ or GHBT_2_ films turned purple upon film removal, indicating UV exposure. Cards covered by the GHBT_2_‐CD film showed an attenuated color change, reflecting improved UV blocking capability. Cards covered with the GHBT_6_‐CD film retained their original color, demonstrating superior UV blocking properties consistent with the UV–vis transmittance spectra.

### 2.4 Thermochromic Properties and Information Encryption Functionality

**FIGURE 5:**
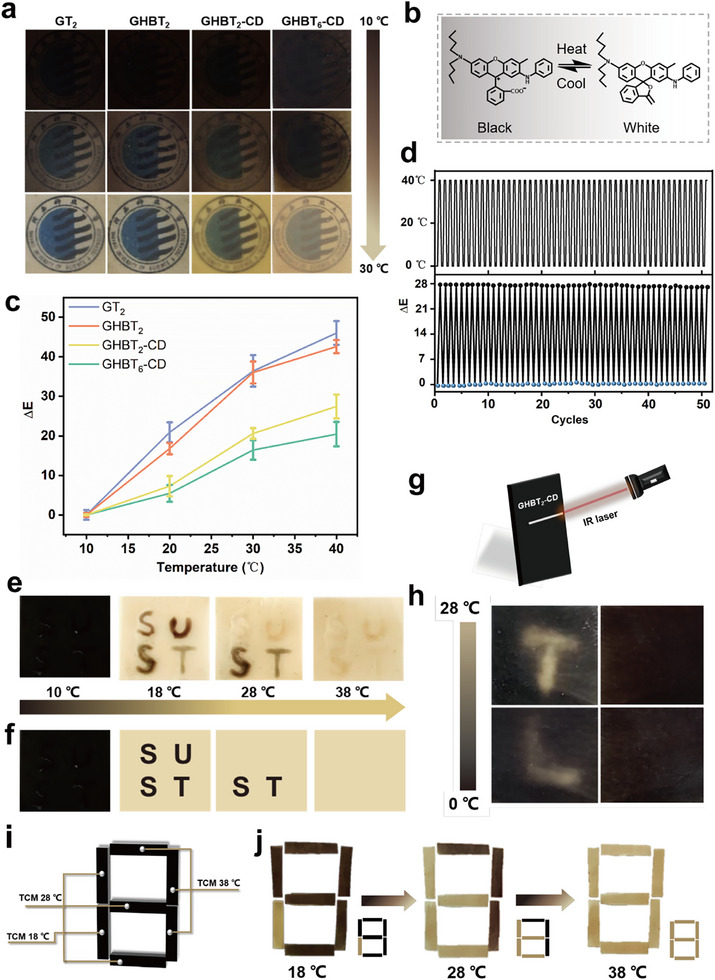
Thermochromic behavior and single‐modal information encryption: a) Temperature‐dependent optical images (10°C–30°C) demonstrating the reversible thermochromism of films. b) Schematic illustration of the thermochromic mechanism within TCMs. c) Total color difference (ΔE) versus temperature (10°C–40°C) for the films. d) Cyclic stability of the GHBT2‐CD film evaluated through color difference over 50 thermal cycles (10°C ↔ 40°C). e) Encrypted “SUST” pattern written on a GHBT_2_‐CD film (containing 18°C TCMs) using inks responsive at 28°C (“SU”) and 38°C (“ST”). f) Schematic illustrating the writing process for the encrypted pattern. g) Schematic of infrared laser writing on a GHBT_2_‐CD film. h) Optical images showing the appearance and disappearance of laser‐written letters “T” and “L” upon heating (0°C–28°C) and cooling. i) Schematic design of a digital encryption system using films with different thermochromic transition temperatures (18°C, 28°C, 38°C) assembled to display the numeral “8”. j) Optical images showing the thermochromic films displaying the numerals “9”, “7”, and “8” at 18°C, 28°C, and >38°C, respectively.

Optical images demonstrate the reversible transparency and color transitions of the films containing TCMs with increasing temperature (10°C–30°C) (Figure 5a; Video S1). As the temperature rises from 10°C to 30°C, all films shift from opaque black to transparent due to structural changes of the thermochromic dyes within the TCMs. Below the solvent's phase transition temperature, the internal thermochromic dyes contact the color developer, gain electrons, and open their lactone ring, producing the opaque black state. At or above this temperature, solvent melting causes dye protonation (electron loss) and lactone ring closure, resulting in a colorless state [51]. However, the GHBT_2_‐CD film retains a persistent yellow hue at 30°C due to n→π^*^ transitions (400–500 nm absorption) from the Schiff base bonds (C═N) formed between Da‐β‐CD and gelatin [52]. Higher TCM content in GHBT_6_‐CD further reduces transparency.

The total color difference (ΔE) versus temperature plots (Figure 5c) show that the GT_2_ film exhibits the maximum ΔE (46.1) across temperatures, indicating superior color reversal. In contrast, the GHBT_2_‐CD films show minimal ΔE variation (27.4), primarily attributed to the persistent light‐yellow tint imparted by the Schiff base bonds, reducing the overall color difference. The GHBT_6_‐CD film, rendered opaque by its high TCM content, demonstrates the smallest ΔE change with temperature.

Based on its optimal thermochromic‐mechanical balance, the GHBT_2_‐CD film (containing 2 wt.% TCMs) was selected for durability assessment. After 50 thermal cycles between 10°C and 40°C, the film demonstrated excellent cyclic stability, with ΔE values showing negligible variation (<5%) in both the cold (10°C) and hot (40°C) states (Figure 5d).

The reversible thermochromism enables surface pattern regulation via localized thermal stimulation. Infrared (IR) laser irradiation achieves localized heating, inducing the thermochromic transition and allowing pattern writing on the film surface. As depicted in Figure 5g, scanning an IR laser across the GHBT_2_‐CD film raises the temperature along its path, resulting in the appearance of the letters “T”(Video S2) and “L”. Given the reversibility of the effect, the displayed “T” and “L” patterns disappear when the film cools below 28°C.

To demonstrate advanced information encryption, TCMs with distinct transition temperatures (18°C, 28°C, 38°C) were incorporated. Solutions containing 28°C and 38°C TCMs were used as ink. The letters “SU” and “ST” were written onto the surface of a GHBT_2_‐CD film containing 18°C TCMs (28°C thermochromic ink for “SU”, 38°C thermochromic ink for “ST”, Figure 5e; Video S3), creating an encrypted coating layer. At 10°C (below all transition points), both the film and the encrypted coating appeared black, displaying no information. Heating to 18°C caused the underlying GHBT_2_‐CD film to turn yellow, revealing the encrypted message “SUST”. Further heating above 28°C caused the “SU” letters (written with 28°C ink) to change color, while “ST” (written with 38°C ink) remained black. Heating to 40°C rendered the entire encrypted coating yellow, causing the information to disappear. Cooling back to 10°C restored the black state. This sequential, reversible color‐switching enables multilevel encryption. Similarly, patterned films were fabricated using TCMs with transition temperatures of 18°C, 28°C, and 38°C (Figure 5h). As depicted in Figure 5i, at 18°C, only the film containing 18°C TCMs turned yellow, leaving dark areas forming the digit “9”. When the temperature rose to 28°C, films containing both 18°C and 28°C TCMs turned yellow, resulting in dark areas forming the digit “7”. Above 38°C, all films turned yellow, causing the yellow areas to form the digit “8”. This principle allows complex patterns to display distinct information at different temperatures for effective information encryption.

### 2.5 Dual‐Modal Information Encryption Based on Fluorescence and Thermochromism

**FIGURE 6:**
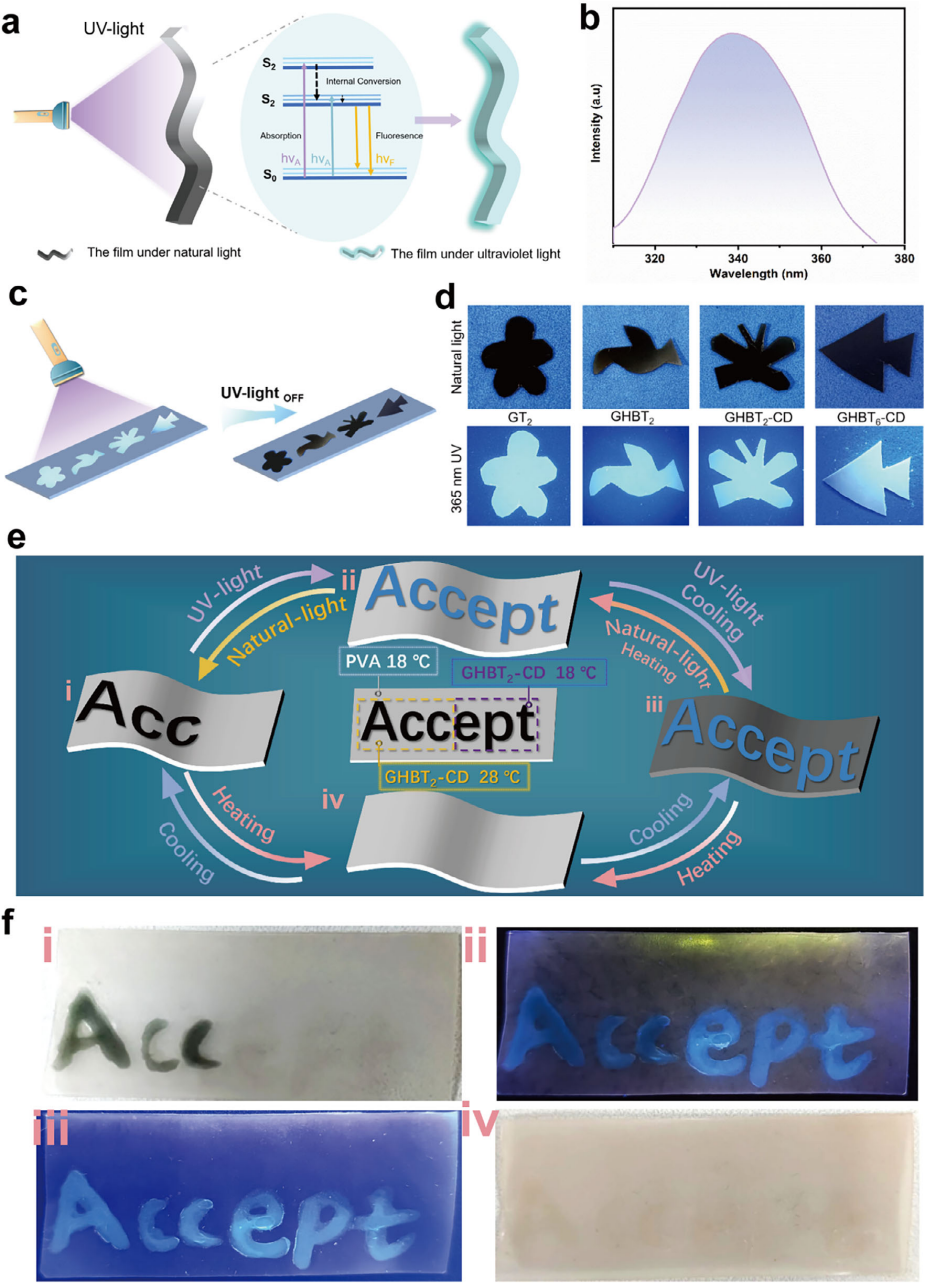
Fluorescence properties and dual‐modal information encryption: a) Proposed mechanism for the intrinsic fluorescence of GHBT_2_‐CD films arising from aromatic amino acids under UV excitation. b) Fluorescence emission spectrum of the GHBT_2_‐CD film under 280 nm excitation, showing a peak at 340 nm. c) Schematic illustrating the four‐state response modulated by temperature and UV illumination using patterned GHBT‐CD solutions with distinct thermochromic transition temperatures. d) Demonstration of the four‐state response: Thermochromic GHBT‐CD solutions patterned as “Acc” (28°C TCMs) and “ept” (18°C TCMs) on a polyvinyl alcohol (PVA) substrate containing 18°C TCMs. e) Schematic design of patterned films (GT_2_, GHBT_2_, GHBT_2_‐CD). f) Corresponding photographs under white light (left) and UV 365 nm illumination (right), demonstrating reversible thermochromic and fluorescent pattern switching.

Gelatin possesses inherent fluorescence, primarily arising from aromatic amino acids (e.g., tryptophan, tyrosine, phenylalanine), which offers potential for expanding applications. UV excitation promotes electrons within these residues from the ground state (S_0_) to excited singlet states (S_1_ or S_2_). Subsequent radiative relaxation from S_1_ to S_0_ produces characteristic fluorescence emission at 340 nm [53]. Consistent with this mechanism, GHBT_2_‐CD films exhibit a distinct emission peak at 340 nm under 280 nm excitation (Figure 6b; Figure S6), confirming the wavelength stability required for optical encryption. Photographs taken under natural and 365 nm UV light (Figure 6d) provide direct visual confirmation of the fluorescence in patterned films.

Combining this intrinsic fluorescence with TCM thermochromism, we designed a dual‐modal (fluorescence/temperature), four‐state information encryption system (Figure 6e,f). This system comprises a polyvinyl alcohol (PVA) substrate embedded with 18°C thermochromic TCMs. Specific patterns were applied: “Acc” written with a modified gelatin solution containing 28°C TCMs, and “ept” written with a solution containing 18°C TCMs. This yields four distinct information states: (1) At 25°C under natural light: since the temperature exceeds the transition points of the substrate (18°C) and the “ept” coating (containing 18°C TCMs), they are rendered colorless. Consequently, only the black “Acc” letters (writing with the solution containing 28°C TCMs, below transition) are visible against the colorless substrate background, displaying “Acc” (Figure 6f‐i). (2) At 25°C under 365 nm UV light: The substrate remains colorless. The “Acc” letters (28°C TCMs) appear black, while the “ept” letters (18°C TCMs) exhibit blue‐green fluorescence, forming the complete fluorescent message “Accept” (Figure 6f‐ii). (3) At 10°C under 365 nm UV light: The substrate cools below 18°C transition, turning black. The “Acc” letters (28°C TCMs, above transition) become colorless, while the “ept” letters (18°C TCMs, below transition) fluoresce blue‐green. Therefore, the fluorescent “Accept” message remains visible against the black substrate (Figure 6f‐iii). (4) At temperatures ≥28°C under natural light: The temperature exceeds the transition of the “Acc” coating (28°C TCMs), rendering it colorless. The substrate (18°C TCMs) is also colorless. Consequently, no distinct message is displayed (Figure 6f‐iv). This encryption system integrates fluorescence and thermochromism to provide enhanced security. Its dual‐validation mechanism, combining fluorescence with reversible thermochromic responses, renders counterfeiting significantly more challenging than in single‐modal systems. The system also enables dynamic, user‐interactive authentication: thermal stimuli, such as heating or laser irradiation, trigger reversible color changes, while the fluorescence signal remains a stable reference, thereby improving reliability through multi‐step verification. Furthermore, the gelatin‐based matrix offers a safer and more eco‐friendly alternative to systems utilizing toxic solvents or non‐degradable polymers. These attributes make the system particularly suitable for high‐security applications such as anti‐counterfeiting labels for food, pharmaceuticals, and cosmetics, where both security and material safety are crucial.

### 2.6 Temperature Regulation Properties of Modified Gelatin‐Based Films

**FIGURE 7:**
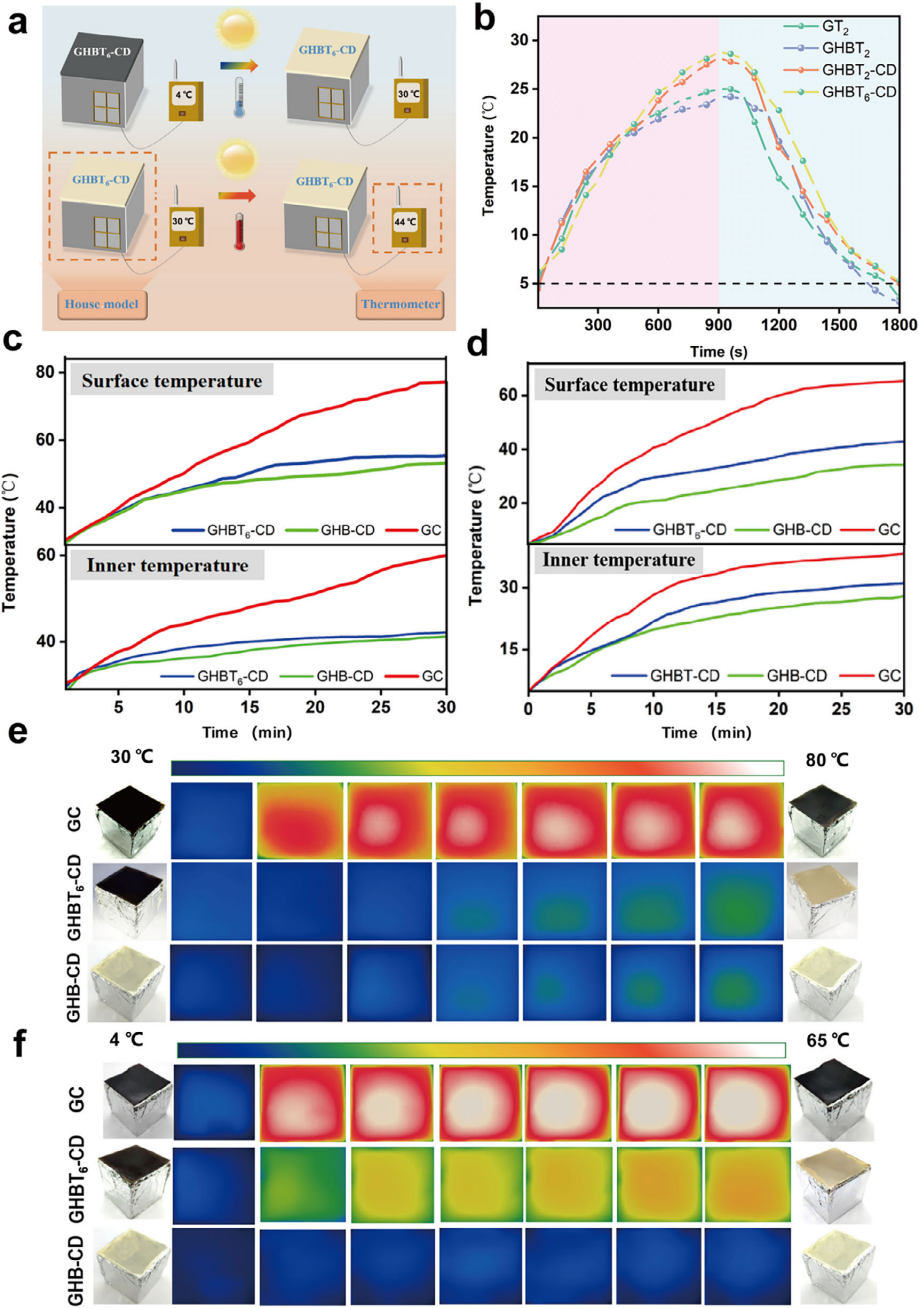
Temperature regulation performance: a) Schematic of the model house setup used for temperature regulation experiments under simulated cold (4°C) and hot (30°C) ambient climates. b) Photothermal heating and subsequent cooling curves of the GHBT_6_‐CD film and control films under “1 sun” irradiation (red shaded region, 4°C ambient) and after irradiation cessation (blue shaded region, 4°C ambient). c) Internal and surface temperatures of model houses covered with different films under “1 sun” irradiation at 30°C ambient. d) Internal and surface temperatures of model houses covered with different films under “1 sun” irradiation at 4°C ambient. e) Photographs and corresponding infrared thermal images of model houses at 30°C ambient after 30 min of “1 sun” irradiation. f) Photographs and corresponding infrared thermal images of model houses at 4°C ambient after 30 min of “1 sun” irradiation.

Leveraging their thermochromic behavior, the films act as intelligent thermal regulators. In low‐temperature environments (<28°C), the organic solvent within TCMs solidifies, enabling the leuco dye and developer to form a conjugated structure, rendering the film black and maximizing solar absorption for heating. Conversely, at temperatures exceeding the solvent's melting point (>28°C), the film transitions to pale yellow, reducing solar absorptivity to prevent overheating [54]. Concurrently, the solid‐liquid phase transition of the solvent absorbs and releases latent heat, forming a thermal buffer layer that delays temperature change rates. To quantify performance, films were applied to a model house, with temperatures monitored via thermocouples (Figure S7). Under “1 sun” irradiation at 4°C ambient (Figure 7b), the GHBT_6_‐CD film (6 wt% TCMs) reached approximately 28.6°C within 900 s. Upon removing irradiation, modified films exhibited delayed cooling, with GHBT_6_‐CD film showing optimal performance (cooling to 5°C after 900 s), attributed to extended latent heat release at higher TCM loadings.

To validate the effectiveness of the GHBT_6_‐CD film under extreme climates, model houses covered with GC (control), GHBT_6_‐CD, and GHB‐CD (gelatin film without TCMs)were subjected to “1 sun” irradiation for 30 min (Figure 7a). Under hot climate conditions (30°C ambient; Figure 7c,e), the GC film‐covered house exhibited severe overheating (surface: 77°C, internal: 60°C), while GHBT_6_‐CD and GHB‐CD films maintained significantly lower temperatures (surface <56°C, internal <42°C) due to thermochromically induced light‐yellow coloration reducing solar absorptivity. Conversely, in cold climates (4°C ambient; Figure 7d,f), the GC film‐covered house reached a surface temperature of 65°C with an internal temperature rise of ΔT = 30.7°C (from 4°C to 34.7°C). The GHBT_6_‐CD film showed moderated heating (surface: 43°C, internal: 31°C; ΔT = +27°C), while the GHB‐CD film demonstrated the lowest internal gain (ΔT = +24°C, from 4°C to 28°C). Infrared images (Figure 7e,f) visually confirmed the temperature differences.

### 2.7 Recyclability and Degradability of Modified Gelatin‐Based Films

**FIGURE 8:**
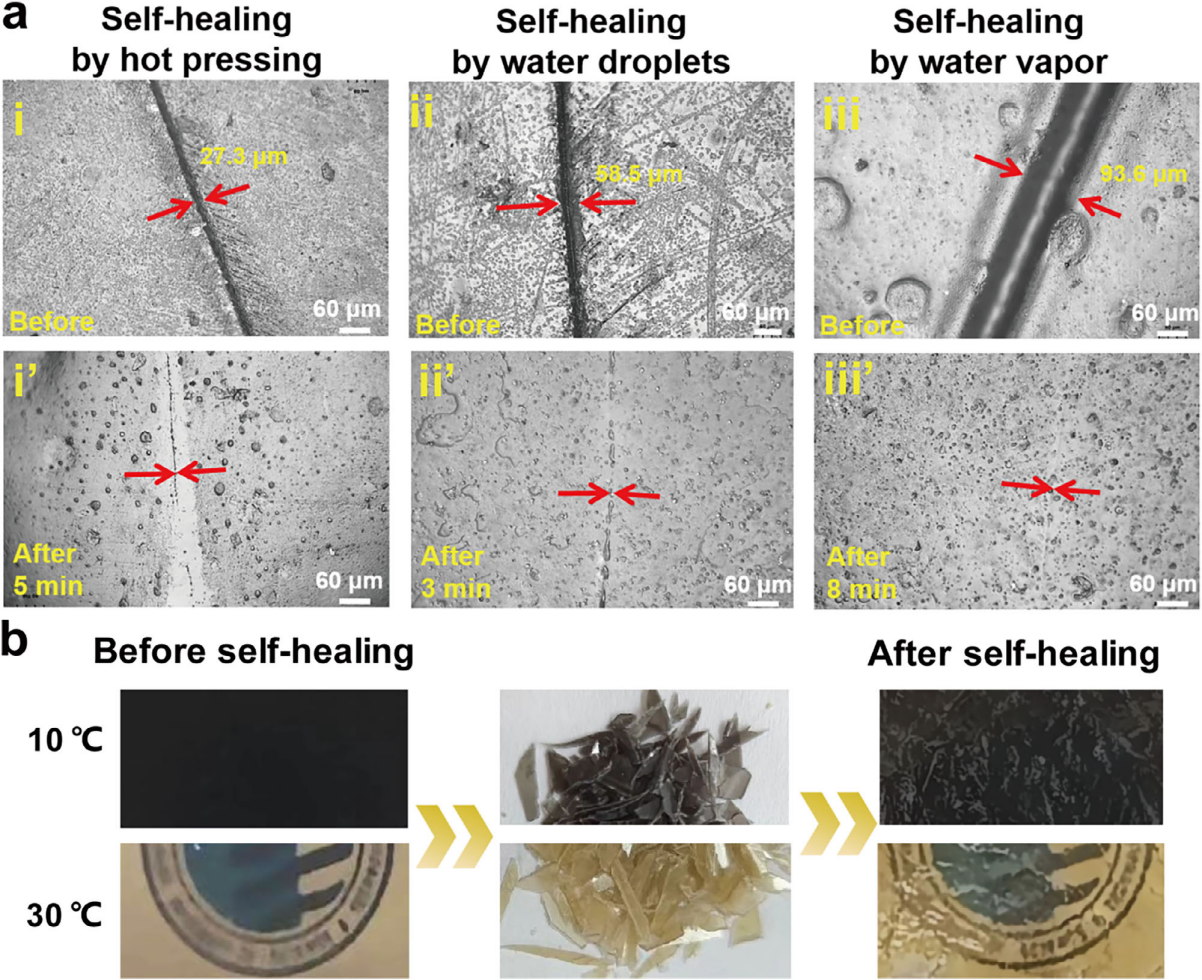
Recyclability and self‐healing properties: a) Ultra‐depth microscopy images of the GHBT_2_‐CD film surface showing scratches before (i–iii) and after (i'–iii') healing via different stimuli: hot pressing (70°C, 10 min), water immersion (5 min), and water vapor treatment (8 min). b) Photographs demonstrating the physical reprocessability of the GHBT_2_‐CD film: original film, crushed fragments, and the reformed film after hot pressing at 65°C for 45 min, shown at different temperatures.

The self‐healing performance, evaluated by ultra‐depth microscopy (Figure 8a), exhibited distinct efficiency depending on the applied stimulus. Hot‐pressing at 70°C for 10 min achieved a healing efficiency 91.3%, though the scratch remained faintly visible. The elevated temperature promoted the reversible cleavage and reformation of imine (Schiff base) bonds while enhancing polymer chains' mobility and hydrogen bond reorganization, thereby effectively repairing microdamage. Water immersion for 5 min yielded a higher efficiency of 93.4%, as water plasticization and the abundant hydrophilic groups facilitated the rapid reorganization of hydrogen bonds. Notably, water vapor treatment for 8 min led to near‐complete scratch erasure with 99.2% efficiency, underscoring the optimal molecular mobility under this condition. The synergistic interactions between dynamic Schiff base bonds and hydrogen bonds primarily endow the material with rapid, efficient, and stimulus‐responsive self‐healing capability. This dynamic network, originating from the cooperative design of HBPG and Da‐β‐CD, not only enables self‐repair but also integrates multiple advanced functions—including optical encryption, photothermal regulation, biodegradability, and antibacterial activity—within a single gelatin‐based system. Furthermore, the network ensures robust reprocessability and functional adaptability, highlighting the material's significant potential for applications in intelligent packaging, anti‐counterfeiting, and thermal management [55, 56].

The sustainability of the films, encompassing end‐of‐life recyclability, self‐repair, and controlled degradation, is critical. Physical reprocessability was assessed by crushing GHBT_2_‐CD films into fragments and reforming them into visually uniform, intact sheets via hot pressing at 65°C for 45 min (Figure 8b). The reformed sheets showed no significant alteration in thermochromic performance, confirming excellent recyclability. This capability originates from dual dynamic bonds within the film structure: reversible Schiff base linkages that undergo exchange upon heating and hydrogen bonding capable of reorganization at room temperature, enabling efficient, mild, low‐temperature reprocessing.

**FIGURE 9:**
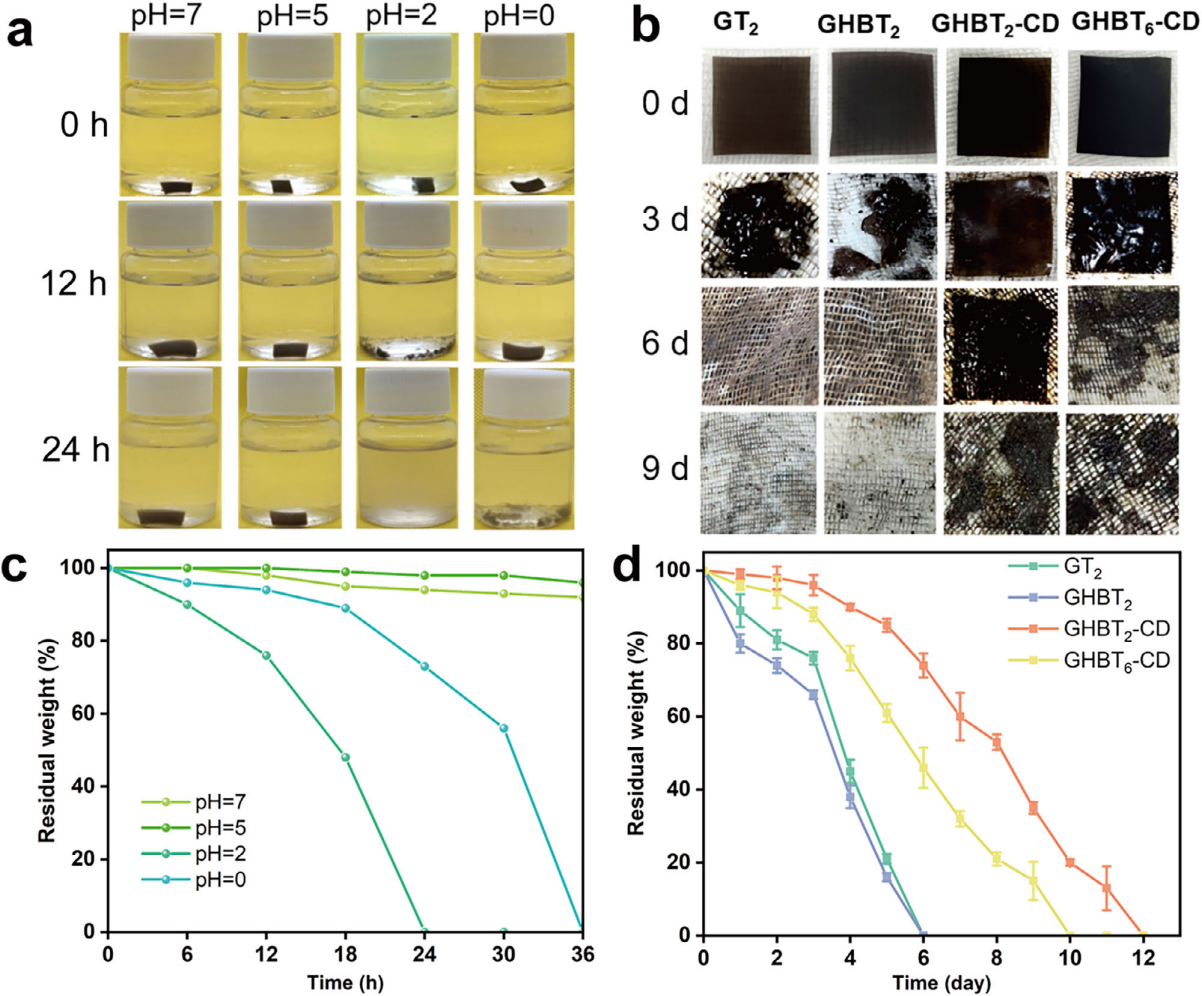
Degradation properties: a) Photographs showing the degradation progression of the GHBT_2_‐CD film over time at different pH values (pH = 0, 2, 5, 7). b) Photographs showing the biodegradation progression of GT_2_, GHBT_2_, GHBT_2_‐CD, and GHBT_6_‐CD films over time in activated sludge. c) Quantitative mass change of the GHBT_2_‐CD film during degradation over time at different pH values. d) Quantitative mass change of the films during biodegradation in activated sludge over time.

Chemical degradability under different pH conditions revealed strong pH dependence (Figure 9a,c). Rapid, complete degradation occurred within 24 h at pH = 2 due to acid‐catalyzed Schiff base hydrolysis. Degradation slowed unexpectedly at pH = 0, potentially due to excessive protonation stabilizing chemical bonds or altering hydrolysis kinetics [57, 58, 59].

Biodegradability was assessed by burying films in activated sludge and monitoring their mass loss over time [60, 61, 62] (Figure 9b,d). The non‐crosslinked GT_2_ and GHBT_2_ films exhibited rapid mass loss (>90% within 5 days) and were fully degraded by day 6. In contrast, the crosslinked GHBT_2_‐CD and GHBT_6_‐CD films degraded more slowly, retaining 85% and 61% of their mass after 5 days, respectively; GHBT_2_‐CD required 12 days for complete degradation. This delay is attributed to the crosslinked 3D network hindering the diffusion of water and microbes, as well as the presence of antimicrobial aldehyde groups that inhibit microbial activity. Importantly, despite the initial delays, the crosslinked films ultimately degraded completely. This demonstrates that the crosslinked network and antimicrobial components regulate the degradation rate without preventing final biodegradation. Therefore, the system reaches a balance between offering antibacterial protection during use and ensuring complete environmental breakdown afterward. As a result, GHBT_2_‐CD films achieve an effective combination of reprocessability, self‐healing, and tunable degradation, which is essential for sustainable advanced materials.

## 3 Conclusion

This study successfully fabricated multifunctional gelatin‐based films through synergistic integration of hyperbranched polyglycerol (HBPG), dialdehyde β‐cyclodextrin (Da‐β‐CD), and thermochromic microcapsules (TCMs). Structural characterization confirmed covalent Schiff base bonds and hydrogen bonding reorganization, endowing the optimized films with superior mechanical strength (28.7 MPa), exceptional UV‐shielding (>97%), and complete antimicrobial efficacy. The resulting material integrates three harmonized functionalities: (1) Programmable Security: TCMs enable temperature‐gated encryption (“SUST” decryption), IR laser writing (“T/L”), and numeral switching (9→7→8), while dual‐modal fluorescence‐thermochromism creates four‐state displays (“Accept” message); (2) Self‐Regulating Thermal Management: The films autonomously switch between photothermal‐heating (black state <28°C, ΔT = +27°C) and heat rejection modes (yellow state >28°C, surface <56°C), buffered by TCM phase‐change; (3) Closed‐Loop Sustainability: dynamic Schiff base/hydrogen bonding enable >91% self‐healing (water/heat/vapor), mild reprocessing (65°C), and tunable degradation (24 h at pH = 2; 12‐day biodegradation). This work lays the foundations for the development of next‐generation multifunctional materials that offer both high performance and environmental protection.

## 4 Experimental Section

Gelatin (gel strength:∼100 g Bloom), glycerol, sodium periodate (NaIO_4_), maleic anhydride, and β‐cyclodextrin (β‐CD) were purchased from Shanghai McLean Biochemical Technology Co., Ltd. Thermochromic microcapsules (TCMs, melamine‐formaldehyde resin shells, with different chromatic transition temperature: 18°C, 28°C, and 38°C, black to colorless) were obtained from Shengshi New Material Co., Ltd. Activated sludge (viable count ≥ 1×10⁹ CFU/g; operational tolerance: 10°C–45°C, pH 6.0–9.0) was supplied by Shandong Dabu Biotechnology Co. Ltd. All chemicals were of analysis grade and used as received.

Da‐β‐CD was synthesized via sodium periodate oxidation following a modified literature procedure [31]. Briefly, β‐CD (10.0 g) was dissolved in deionized water (120 mL) at 55°C under stirring. After cooling to 35°C, NaIO_4_ (14.4 g) was added gradually until fully dissolved. The reaction mixture was acidified to pH 3.0 using 0.1 m HCl and stirred for 12 h at ambient temperature under dark conditions (aluminum foil‐wrapped vessel). The crude product was purified by dialysis (MD44 membrane, MWCO 500 Da) against deionized water for 48 h, with water changes every 6 h. The purified Da‐β‐CD was lyophilized at −40°C under reduced pressure for 48 h to obtain a white powder.

Hyperbranched polyester (HBPG) was synthesized via polycondensation of glycerol and maleic anhydride, adapted from the literature [41]. Glycerol (4.9 g) and maleic anhydride (4.7 g) were charged into a nitrogen‐purged three‐neck flask equipped with a mechanical stirrer. Under continuous nitrogen flow, the mixture was stirred at 65°C for 1 h to form a homogeneous solution. Polycondensation reaction proceeded at 100°C for 2 h, followed by 120°C for 3 h, yielding a light‐yellow viscous HBPG product.

Films were prepared by solution casting (Figure 1). Gelatin (2.0 g) was dissolved in deionized water (21 mL) at 60°C under stirring. HBPG (0.8 g) and Da‐β‐CD (0.1 g) were added, and the mixture (designated GHB‐CD) was stirred at 45°C for 1 h. TCMs (2 wt% or 6 wt% relative to gelatin) were then incorporated to form GHBT_2_‐CD or GHBT_6_‐CD mixtures, respectively (Table 1). The mixtures were cast onto polytetrafluoroethylene molds and dried at 25°C for 48 h. Control films were prepared identically: GT_2_ (gelatin, 20 wt% glycerol, 2 wt% TCMs without HBPG and Da‐β‐CD) and GHBT_2_ (gelatin, HBPG, 2 wt.% TCMs without Da‐β‐CD).

**TABLE 1:** Composition of gelatin‐based films.

Sample	Gelatin (g)	Glycerol (g)	HBPG (g)	Da‐β‐CD (g)	TCMs (g)
GT_2_	2.0	0.60	0	0	0.04
GHBT_2_	2.0	0	0.80	0	0.04
GHBT_2_‐CD	2.0	0	0.80	0.10	0.04
GHBT_6_‐CD	2.0	0	0.80	0.10	0.06

Two control films were prepared to evaluate thermal regulation performance. The non‐photothermal GHB‐CD film was fabricated by dissolving gelatin (2.0 g) in deionized water (21 mL) at 60°C, followed by adding HBPG (0.8 g) and Da‐β‐CD (0.1 g). The mixture was stirred at 45°C for 1 h, cast into a Petri dish, and dried for 48 h. The photothermal control (GC film) was prepared by dissolving gelatin (2.0 g) in deionized water (21 mL) at 60°C. After stirring for 1 h, carbon black (0.6 g) and glycerol (0.4 g) were added, and stirring continued at 45°C for 1 h. The solution was cast and dried for 48 h.

Fourier‐transform infrared (FTIR) spectroscopy was performed on a Bruker Vertex70 spectrometer (Germany) at 4 cm^−1^ resolution (4000–400 cm^−1^). Crystalline structures were analyzed using X‐ray diffraction (XRD; Bruker D8 Advance, Germany) with 2θ scans from 5° to 60°. Elemental composition and chemical bond were characterized by X‐ray photoelectron spectroscopy (XPS; KRATOS Ultra DLD, UK), with the C 1s peak referenced to 284.8 eV.

The aldehyde content of Da‐β‐CD was determined via hydroxylamine hydrochloride titration using methyl orange as the indicator. In this method, aldehyde groups react with hydroxylamine hydrochloride to release an equimolar amount of HCl, which is then determined by back‐titration with a standardized NaOH solution. The aldehyde content was calculated as:

(1) $$\mathrm{Aldehyde}\;\mathrm{content}\;\left(\mathrm{mmol}\;g^{-1}\right)=\frac{\left(V_{t}-V_{0}\right)C_{\mathrm{NaOH}}\times 1000}{m}$$

where V_t_ and V_0_ are the NaOH volumes (mL) consumed in the sample and blank titrations, respectively, C_NaOH_ is the molar concentration of the NaOH solution, and m (g) is the mass of the Da‐β‐CD sample. All measurements were performed in triplicate.

Prior to testing, the films were equilibrated at 25°C and 50% relative humidity for 48 h. Dumbbell‐shaped specimens were prepared, and their thickness was measured at five points along the gauge length using a digital micrometer. Tensile tests were performed using a universal testing machine with a 20 mm gauge length and a crosshead speed of 5 mm/min. The toughness was determined by calculating the area under the stress‐strain curve. For each formulation, five replicate specimens were tested.

Thermal stability was assessed via thermogravimetric analysis (TGA; TA Instruments Q500) under N_2_ atmosphere (25°C–650°C, 10°C·min^−1^).

Surface and cross‐sectional morphologies were examined using a scanning electron microscope (SEM; TESCAN, Czech Republic). Bacterial adhesion on film surfaces was visualized by field‐emission SEM (FEI Verios 460, USA) at 2 kV. 3D surface topography was characterized using a Hirox RH‐2000 super‐depth microscope (Japan).

Water vapor permeability (WVP) was measured at 38°C and 90% relative humidity using a W3/060 tester (Jinan Lance), following ASTM E96 (triplicates).

The UV–vis spectra (200–800 nm) of the films were obtained using an Agilent Cary 100 spectrophotometer. The UV‐blocking efficiency was calculated according to the standard method using the following equation:

(2) $$\mathrm{UV}-\mathrm{blocking}\;\mathrm{rate}\;\left(\%\right)=\left[1-\frac{1}{L}\int_{\lambda_{\min}}^{\lambda_{\max}}T\left(\lambda \right)d\lambda \right]\times 100\%\;$$

where L = λ_max_−λ_min_ is the wavelength bandwidth, and T(λ) is the transmittance at wavelength λ.

Total color difference (ΔE) was quantified using a CS‐220 colorimeter (Hangzhou CHN Spec Technology). Photothermal responses were evaluated under simulated sunlight (1 kW m^−2^) from a xenon lamp (Perfectlight PLS‐FX300HU), with temperatures monitored by a VICTOR 6801 thermometer and TESTO 872 thermal camera. Detailed optical protocols are provided in Supplementary Information.

The minimum bactericidal concentration (MBC) was defined as the lowest antimicrobial concentration that reduces the viable microbial population by ≥99.9%. To determine the MBC, bacterial suspensions (OD_600_ ≤ 0.5) were incubated with GHBT_2_‐CD at concentrations of 0, 3.5, 7.0, 10.5, and 14.0 mg/mL in LB medium (20 mL containing 20 µL of bacterial suspension) for 6 h. Subsequently, the suspensions were serially diluted 1000‐fold. A 100 µL aliquot of each dilution was spread onto nutrient agar plates. After incubation at 37°C for 12 h, the colony‐forming units (CFU) were counted. The results were expressed as log_10_(CFU/mL). All experiments were performed in triplicate.

Antibacterial efficacy against *S. aureus* (ATCC 6538) and *E. coli* (ATCC25922) was evaluated according to a modified ISO 22196 standard. Bacterial suspensions (10⁷ CFU mL^−1^ in PBS) were incubated with films (1 × 1 cm^2^) at 37°C for 24 h. Serial dilutions were plated on tryptic soy agar, incubated 24 h at 37°C, and colonies enumerated. The antibacterial rate (*R*, %) was calculated as:

(3) $$R=\frac{N_{0}-N_{x}}{N_{0}}\times 100\%$$

where *N*_0_ and *N*_x_ represent control and sample colony counts (CFU mL^−1^), respectively (triplicate measurements).

Bacterial suspensions (OD_600_ ≈0.5) were incubated with film samples in Erlenmeyer flasks at 37°C under static conditions for 6 h. The bacteria were then harvested by centrifugation at 6000 rpm. The pellet was fixed overnight at 4°C with 2.5% glutaraldehyde in PBS (pH 7.4), washed with PBS, and dehydrated through a graded ethanol series (30, 50, 70, 80, 90, 95, and 100 v/v%). A drop of the bacterial suspension in absolute ethanol was placed on a polished silicon wafer, air‐dried overnight in a desiccator, and observed by SEM.

*S. aureus* and *E. coli* were cultured overnight at 37°C. Cells were harvested by centrifugation (4000 rpm, 10 min), washed twice with PBS, and fixed in 2.5% glutaraldehyde (PBS) for 2 h at 4°C. After washing, the samples were post‐fixed with 1% osmium tetroxide for 1 h at 4°C, followed by dehydration in a graded ethanol series (30%–100%). The dehydrated samples were embedded in epoxy resin. Ultrathin sections (70 nm) were cut using an ultramicrotome, mounted on copper grids, and stained with 2% uranyl acetate (15 min) and 1% lead citrate (10 min). Observations were made under a TEM at 80 kV.

Film samples were incubated with bacterial suspensions (OD_600_ ≈0.5) at 37°C for 6 h. After incubation, the suspensions were centrifuged, and the supernatant was collected. The release of nucleic acids and proteins due to membrane damage was quantified by measuring the absorbance of the supernatant at 260 and 280 nm using a UV–vis spectrophotometer. The surface charge of bacteria after treatment with film samples was determined using a zeta potential analyzer. Bacterial suspensions treated as described above were used for measurement.

The biofilm inhibition activity of the film samples was assessed quantitatively via the crystal violet staining method. Suspensions of *E. coli* and *S. aureus* (adjusted to 1 × 10⁸ CFU·mL^−1^ in LB medium) were added to a 48‐well plate (400 µL per well) and incubated at 37°C for 48 h to allow biofilm formation, with the culture medium refreshed every 12 h. Subsequently, film samples (10 mm × 10 mm × 0.3 mm) were placed into the wells and co‐cultured with the preformed biofilms for 24 h. After co‐culture, the films and medium were removed. The wells were gently washed three times with PBS and air‐dried. The adherent biofilms were then stained with 0.1 wt.% crystal violet solution (100 µL per well) for 15 min at room temperature, followed by three PBS rinses to remove unbound dye. For quantitative analysis, the bound dye was dissolved with 200 µL of 95 wt.% ethanol per well. The resulting solution was transferred to a 96‐well plate, and its absorbance was measured at 570 nm.

The biofilm inhibition rate (E) was calculated using Equation (4):

(4) $$E=\frac{\left(1-A_{x}\right)}{A_{0}}\times 100\%$$

where A_x_ and A_0_ represent the absorbance at 570 nm for the bacterial suspension treated with the modified film and the control film (original film), respectively. All measurements were performed in triplicate.

Fluorescence excitation/emission spectra were acquired on an Edinburgh FS5 spectrofluorometer. Patterned films for dual‐modal encryption were fabricated by coating polyvinyl alcohol (PVA) substrates (containing 18°C‐responsive TCMs) with thermochromic gelatin solutions (“Acc”: 28°C TCMs; “ept”: 18°C TCMs). Fluorescent responses were characterized under UV (365 nm) and visible light across 10°C–40°C.

For thermal regulation, model houses roofed with GC, GHBT_6_‐CD, or GHB‐CD films were subjected to “1 sun” irradiation (1 kW m^−2^) in simulated cold (5°C) and hot (30°C) environments. Interior temperatures were recorded using K‐type thermocouples, and exterior surfaces were monitored via infrared thermography.

Reprocessability was evaluated by hot‐pressing GHBT_2_‐CD film fragments at 65°C for 15 min, cooled to 25±2°C, and assessing structural integrity and thermochromic performance.

Self‐healing properties were characterized by creating artificial scratches on GHBT_2_‐CD films and applying three treatments: (i) dry heat (65°C, 20 min), (ii) water immersion (25°C, 5 min), and (iii) water vapor exposure (100°C, 8 min). Surface morphology before and after treatment was analyzed using HIROX microscopy. Healing efficiency (η, %) was calculated as:

(5) $$\eta =\frac{L_{0}\cdot W_{0}-L_{t}\cdot W_{t}}{L_{0}\cdot W_{0}}\times 100\%$$

where *L*_0_ and *W*_0_ represent initial crack length and width (µm), and *L*_t_ and *W*_t_ denote residual crack length and width after healing.

Chemical degradation behavior was assessed by immersing GHBT_2_‐CD and GHBT_6_‐CD films in HCl solutions (pH = 0, 2, 5) and pH = 7, at 25 ± 2°C with stirring. Dissolution was monitored hourly until complete degradation.

Biodegradation was conducted by incubating GHBT‐CD films (1×1 cm^2^) in activated sludge (28.0 ± 0.5°C, 80 ± 2% RH) for 12 days. Samples were retrieved at 24 h intervals (days 0–12), washed, dried at 40°C, and weighed. The biodegradation rate (%) was calculated as:

(6) $$\mathrm{Biodegradation}=[\left(M_{0}-M_{t}\right)/M_{0}]\times 100\%$$

where *M*_0_ is the initial dry mass, and *M*_t_ is the mass at time t.

## Conflicts of Interest

The authors declare no conflicts of interest.

## Supporting information




**Supporting File 1**: advs73687‐sup‐0001‐SuppMat.docx.

**Supporting File 2**: advs73687‐sup‐0002‐VideoS1.mp4.

**Supporting File 3**: advs73687‐sup‐0003‐VideoS2.mp4.

**Supporting File 4**: advs73687‐sup‐0004‐VideoS3.mp4.

## Data Availability

The data are available from the corresponding author upon reasonable request.
