# Supplementary material for: Multifunctional Gelatin‐Based Smart Films Integrating Thermochromic Encryption, Temperature‐Regulated Photothermal Management, Reprocessability, and Biodegradability for Sustainable Applications
Source: Adv Sci (Weinh). 2026 Jan 4;13(16):e16955. doi: 10.1002/advs.202516955 (PMC13042584; doi:10.1002/advs.202516955)
Supplement: Supplementary file 1 — Supporting File 1: advs73687‐sup‐0001‐SuppMat.docx. [file ADVS-13-e16955-s002.docx]

**Supporting information**

## Multifunctional Gelatin-Based Smart Films Integrating Thermochromic Encryption, Temperature-Regulated Photothermal Management, Reprocessability and Biodegradability for Sustainable Applications

Yuehong Zhang^a,b*^, Chen Yang^a^, Langlang Dai^a^, Leipeng Liu^a*^, Vijay Kumar Thakur^c*^

^a^College of Bioresources Chemical and Materials Engineering, Shaanxi University of Science and Technology, Xi'an 710021, China.

^b^School of Chemical Engineering and Technology, Tianjin University, Tianjin 300072, China.

^c^Biorefining and Advanced Materials Research Centre, Scotland’s Rural College,

Scotland, Edinburgh EH9 3JG, UK.

E-mail: YH zhang, [yuehong.zhang@sust.edu.cn;](mailto:yuehong.zhang@sust.edu.cn;) LP Liu, [liuleipeng@sust.edu.cn;](mailto:liuleipeng@sust.edu.cn;) VK Thakur, [Vijay.Thakur@sruc.ac.uk](mailto:Vijay.Thakur@sruc.ac.uk)

**

**

**Figure S1.** FTIR spectra of GT_2_, GHBT_2_, GHBT_2_-CD, and GHBT_6_-CD films.


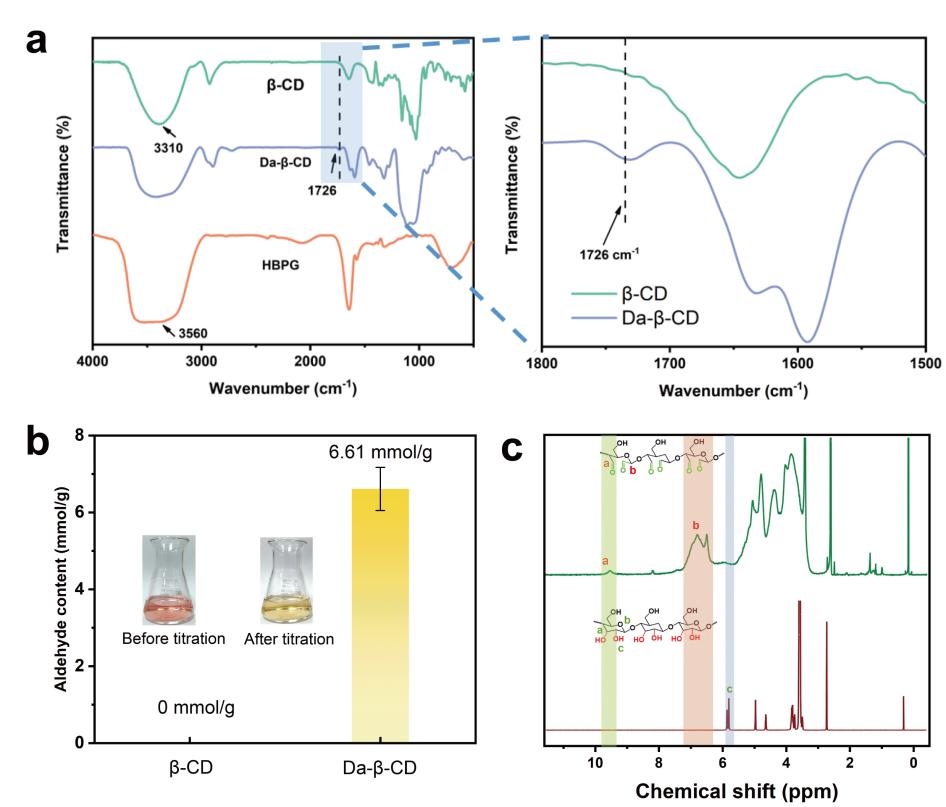


**Figure S2.** (a) FTIR spectra of HBPG, β-CD, and Da-β-CD. (b) Photos of Da-β-CD solution before and after hydroxylamine hydrochloride titration and aldehyde content. (c) ^1^H NMR spectra of β-CD and Da-β-CD.


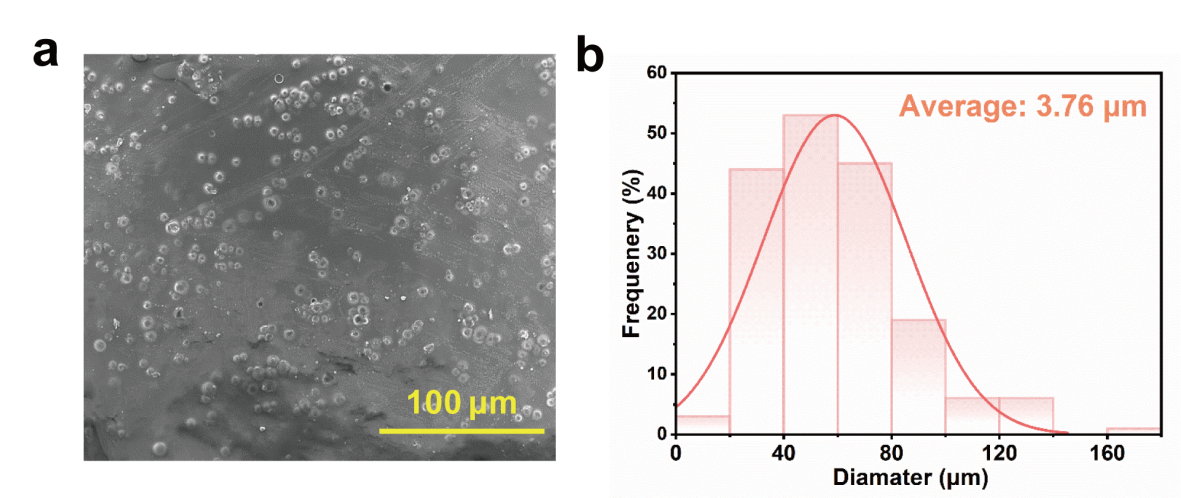


Figure S3. (a) SEM image of the GHBT_6_-CD film surface. (b) Diameter distribution of TCMs measured from SEM images.


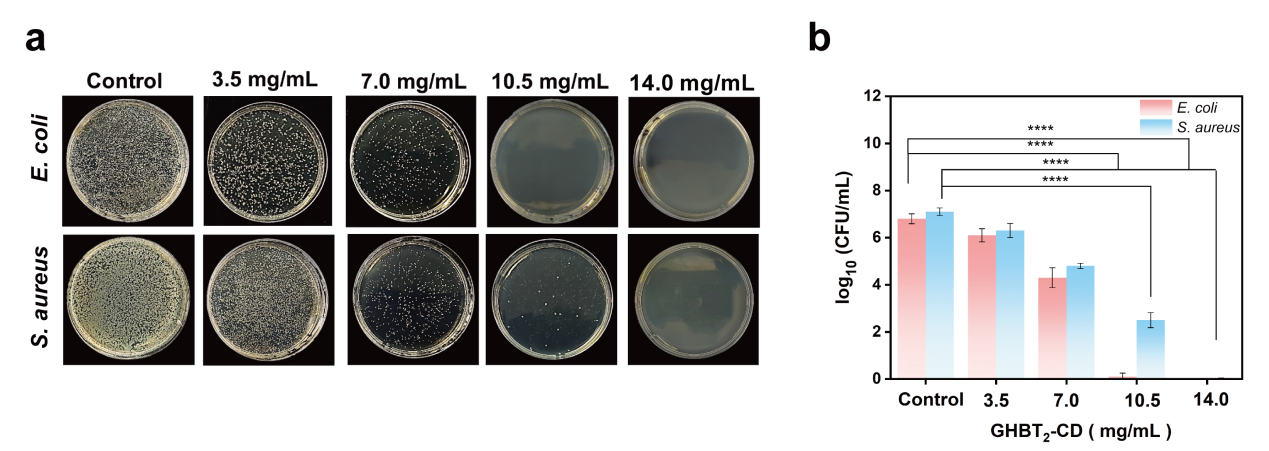


**Figure S4.** (a) Images of bacterial colonies on agar plates after treatment with varying concentrations of GHBT_2_-CD. (b) Quantitative analysis of bacterial viability presented as colony-forming unit (CFU) values.


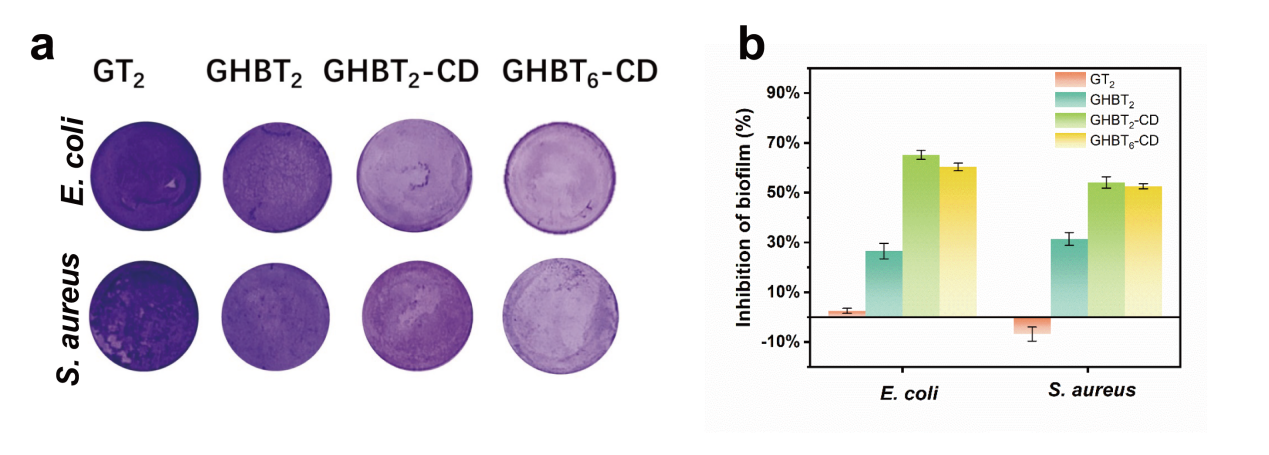


**Figure S5.** (a) Images of crystal violet-stained biofilms. (b) Quantitative measurement of biofilm inhibition performance.





**Figure S6**. Fluorescence emission spectra of various films upon excitation at 280 nm.

**
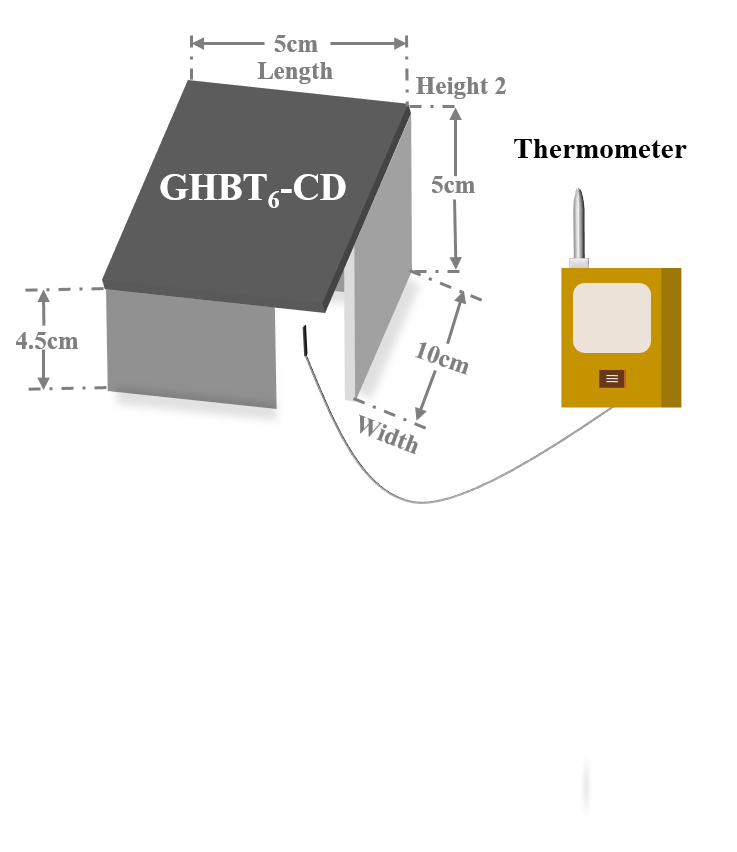
**

**Figure S7.** Schematic illustration of the small house model employed to evaluate thermal regulation performance.

**Table S1** XPS analysis of GT, GHBT, GHBT_2_-CD, and GHBT_6_-CD: C 1s, O 1s, and N 1s binding energies and peak assignments.

| Elements | Assignments | At (%) of different samples | |
| --- | --- | --- | --- |
|  |  | GHBT_2_ | GHBT_2_-CD |
| C 1s | C-C&C-O-C&C-N&C=C | 56.78 | 52.89 |
| C 1s | C-O&C=O&O-C-O&C=N | 28.53 | 35.28 |
| C 1s | O=C-N&-COO^-^ | 5.22 | 13.60 |
| O 1s | C-O&C-O-C | 21.02 | 21.77 |
| O 1s | C=O&O-C-O | 54.09 | 44.21 |
| O 1s | -COO^-^ | 24.89 | 34.02 |
| N 1s | N-H | 53.51 | 24.17 |
| N 1s | N-C | 46.49 | 54.14 |
| N 1s | N=C | / | 21.68 |

Table S2. Functional and performance comparison of the GHBT-CD Film with representative

gelatin-based materials.

|  | Tensile stress (MPa) | UV-Blocking (%) | Antimicrobial (%) | Information encryption | Temperature Regulation | Self-healing | Ref |
| --- | --- | --- | --- | --- | --- | --- | --- |
| GHBT_2_-CD | 28.7 | 97 | 100 (E.coli)  100 (S.aureus) | Dual-Modal Information Encryption | self-adaptive temperature regulation | Highly efficient | This work |
| TP-HBPE-GE-5 | 25.1 | 99.8 | 99.9 (E.coli)  99.9 (S.aureus) | / | / | / | [1] |
| ZnO/HBPSi-GE-5 | 45.8 | 99.6 | 90.6 (E. coli)  89.5% (S. aureus) | / | Cooling only; no heating function | Highly efficient | [2] |
| flexible film | 0.039 | / | / | Single-Modal Information Encryption | / | / | [3] |
| FSG/CS-3% NPs | 18.97 | 65 | 99 (E. coli)  99 (S. aureus) | / | / | / | [4] |
| Gel-MAAs/DS | 5.8 | 100 | / | / | / | / | [5] |
| GCS-3 | 25 | 100 | 100 (E. coli)  99.63 (S. aureus) | / | / | / | [6] |
| PT10:PLA + 10 wt% TCM | 27 | / | / | Single-Modal Information Encryption | self-adaptive temperature regulation | / | [7] |
| TSTM-2 | 10 | / | / | Single-Modal Information Encryption | self-adaptive temperature regulation |  | [8] |
| TCHSC | / | / | / | Single-Modal Information Encryption | self-adaptive temperature regulation | / | [9] |

**References**

1. Y. Zhang, L. Dai, C. Yang, B. Wei, and G. Liao, Green Chem. 2025, 27, 10875.
2. Y. Zhang, L. Dai, and C. Yang, Chem. Eng. J. 2025,169251.
3. W. Zhang, W. Wang, L. Xia, G. Zhou, and J. Wang, Chem. Eng. J. 2025, 164575.
4. J. Chen, K. Huang, X. Zhang, H. Lin, and D. P. Yang, Chem. Eng. J. 2025, 512, 162415.
5. D. Wang, K. Li, J. Zhang, K. Zhou, K. Wang, Y. Wang, S. Yang, S. Wang, P. Wang, and H. Chen, Chem. Eng. J. 2025, 163182.
6. Q. Zhao, X. Huang, L. Qian, N. Sun, J. Yang, J. Wen, H. Li, J. Yang, L. Mo, W. Gao, and Z. Qin, Food Hydrocoll. 2025, 160, 110752.
7. Z. Cheng, Z. Chen, B. Zhao, H. Liao, T. Yu, and Y. Li, Int. J. Biol. Macromol. 2022, 220, 238.
8. B. Xiang, L. Xu, Y. Li, L. Jiao, and R. Zhang, ACS Appl. Polym. Mater. 2024, 6, 12568.
9. Y. Liu, Y. Wu, Y. Ma, P. Wang, B. Yu, X. Pei, S. Liu, and F. Zhou, Chem. Eng. J. 2024, 482, 148837.
